# A119 ACUTE COLONOC PSEUDO-OBSTRUCTION AFTER ENDOSCOPIC RESECTION OF A FULLY CIRCUMFERENTIAL LARGE NON-PEDUNCULATED COLORECTAL POLYP

**DOI:** 10.1093/jcag/gwad061.119

**Published:** 2024-02-14

**Authors:** A Zarrin, S X Jiang, R Gilhotra, N Shahidi

**Affiliations:** The University of British Columbia Faculty of Medicine, Vancouver, BC, Canada; The University of British Columbia Faculty of Medicine, Vancouver, BC, Canada; St. pauls' Hospital, Division of Gastroenterology, Vancouver, BC, Canada; St. pauls' Hospital, Division of Gastroenterology, Vancouver, BC, Canada

## Abstract

**Background:**

Complex large non-pedunculated colorectal polyps (LNPCPs), including those involving the full circumference of the bowel, are now readily managed by minimally invasive endoscopic resection techniques. Established procedure-related adverse events include sedation related adverse events, deep mural injury, clinically significant post-resection bleeding, delayed perforation and serositis. An appreciation for potential procedure-related adverse events is critical to the effective implementation of these techniques.

**Aims:**

To describe a case of acute colonic pseudo-obstruction after endoscopic resection of a fully circumferential LNPCP.

**Methods:**

Case report and review of the literature

**Results:**

A 91-year-old male with a history of atrial fibrillation, chronic kidney disease and suspected McKittrick-Wheelock syndrome underwent successful endoscopic mucosal resection (EMR) of a 100mm fully circumferential LNPCP in the sigmoid colon. Histopathology diagnosed a tubulovillous adenoma with high-grade dysplasia. After elective admission, he suffered from clinically significant post-EMR bleeding requiring 2 units of packed red blood cells. On post-operative day 1 he began to suffer from nausea, abdominal pain and obstipation. On examination, he was visibly distended. Abdominal X-ray revealed extensive gas-filled loops of small and large bowel with the absence of gas in the rectum. He was managed conservatively with nasogastric tube decompression. Total duration of hospital stay was 9 days.

**Conclusions:**

Critical to the continued expansion of minimally invasive endoscopic resection techniques is an awareness for potential procedure-related adverse events. To our knowledge this is the first description of acute colonic pseudo-obstruction after endoscopic resection

**Funding Agencies:**

None

